# Supplementary material for: Chromothripsis during telomere crisis is independent of NHEJ, and consistent with a replicative origin
Source: Genome Res. 2019 May;29(5):737–49. doi: 10.1101/gr.240705.118 (PMC6499312; doi:10.1101/gr.240705.118)
Supplement: Supplemental Material [file supp_gr.240705.118_Supplemental_file_1.zip › contigs/annotated_contigs/DB57/contig.2.DB57_length_269_mean_cov_4.65427509294.docx]

**DB57_length_269_mean_cov_4.65427509294**

```
CATGAAATCAGAAATTTATT|AATGGACAGGATTACGTGTGTGTGTGTGTGTGTG|TGTGTGTGTGTGTGT|GTGTGTGTGTGTGTGTG
                     >chr12:127859429-127859478 - E=4e-15 p=0e+00 >chr7:153733105-1537
AAAATAAACAATATGATTAGTGTTTATTCTTTTTAACAATCCACAAATTGCCTGCTTTTCTCTACTTTTTTTTTGAGATCAAGTCTCAC
33321 + E=3e-108
TCTGTTACCCAGGCTGGAGTACAGTGGGTGTGATCTCAGTGTACTGCAACCTCCGCCTCACAGGTTCAAATGATTCTCCTGCCTCAGCC

TTGCC
```
